# *Mmp13* deletion in mesenchymal cells increases bone mass and may attenuate the cortical bone loss caused by estrogen deficiency

**DOI:** 10.1038/s41598-022-14470-w

**Published:** 2022-06-17

**Authors:** Filipa Ponte, Ha-Neui Kim, Aaron Warren, Srividhya Iyer, Li Han, Erin Mannen, Horacio Gomez-Acevedo, Intawat Nookaew, Maria Almeida, Stavros C. Manolagas

**Affiliations:** 1 Division of Endocrinology and Metabolism, Center for Osteoporosis and Metabolic Bone Diseases, University of Arkansas for Medical Sciences, 4301 W. Markham St. #587, Little Rock, AR 72205-7199 USA; 2 Department of Orthopedic Surgery, University of Arkansas for Medical Sciences, Little Rock, USA; 3 Department of Biomedical Informatics, University of Arkansas for Medical Sciences, Little Rock, USA; 4 The Central Arkansas Veterans Healthcare System, Little Rock, USA

**Keywords:** Osteoporosis, Bone

## Abstract

The protective effect of estrogens against cortical bone loss is mediated via direct actions on mesenchymal cells, but functional evidence for the mediators of these effects has only recently begun to emerge. We report that the matrix metalloproteinase 13 (MMP13) is the highest up-regulated gene in mesenchymal cells from mice lacking the estrogen receptor alpha (ERα). In sham-operated female mice with conditional Mmp13 deletion in Prrx1 expressing cells (*Mmp13*^ΔPrrx1^), the femur and tibia length was lower as compared to control littermates (*Mmp13f.*^/f^). Additionally, in the sham-operated female *Mmp13*^ΔPrrx1^ mice cortical thickness and trabecular bone volume in the femur and tibia were higher and osteoclast number at the endocortical surfaces was lower, whereas bone formation rate was unaffected. Notably, the decrease of cortical thickness caused by ovariectomy (OVX) in the femur and tibia of *Mmp13f.*^/f^ mice was attenuated in the *Mmp13*^*ΔPrrx1*^ mice; but the decrease of trabecular bone caused by OVX was not affected. These results reveal that mesenchymal cell–derived MMP13 may regulate osteoclast number and/or activity, bone resorption, and bone mass. And increased production of mesenchymal cell-derived factors may be important mediators of the adverse effect of estrogen deficiency on cortical, but not trabecular, bone.

## Introduction

During the last 10 years, we and others have elucidated in genetic mouse models that the protective effects of estrogens on trabecular and cortical bone mass are mediated via ERα actions on distinct cell types: hematopoietic and mesenchymal lineage cells, respectively^1,2^. In trabecular bone direct estrogen actions on osteoclast decrease their number by promoting apoptosis. More recently, we showed that this direct effect likely results from decreased expression and activity of mitochondria complex I genes, “oxidative phosphorylation” and respiration (oxygen consumption rate) and it requires Bak and Bax, two members of the Bcl-2 family of proteins that are critical for mitochondrial apoptotic death^3^. In the cortical bone compartment, however, estrogens decrease osteoclast number indirectly by suppressing the expression of pro-osteoclastogenic factors produced by cells of the mesenchymal lineage. In support of this latter mechanism of action, we have also recently shown that in mice with conditional deletion of *Cxcl12* in *Prrx1* cells, the loss of cortical, but not trabecular, bone mass caused by estrogen deficiency is attenuated^4^.

Proteolytic breakdown of extracellular matrix components plays an important role during bone remodeling. Collagen 1 and 2, the most abundant extracellular matrix components of bone and cartilage, are recycled via the activity of matrix metalloproteinase (MMP) family of enzymes. MMP13 is highly expressed in terminally differentiated hypertrophic chondrocytes and osteoblasts. Also, a mutation of the human *Mmp13* gene causes the Missouri variant of spondyloepimetaphyseal dysplasia (SEMD), a disorder characterized by abnormal development and growth of vertebrae and long bones^5^. Studies of mice with global or conditional *Mmp13* deletion have further elucidated the role of this metalloproteinase on the skeleton^6,7^. MMP13 deficiency in chondrocytes alters growth plate architecture and in osteoblasts/osteocytes increases trabecular bone mass^7,8^. Several lines of evidence have implicated metalloproteinase in bone resorption. Indeed, the stimulation of bone resorption by parathyroid hormone (PTH) requires collagenase cleavage of type I collagen^9^. In addition, MMP13 stimulates osteoclast differentiation and activation in breast tumor bone metastases^10^ and is involved in the osteolytic lesions of multiple myeloma^11^. Interestingly, the action of MMP13 in myeloma results from its ability to promote osteoclast fusion by up-regulating the fusogen DC-STAMP, independently of its enzymatic activity.

Notably, MMP13 mRNA and protein increase in the osteoblasts of ovariectomized rats; and inhibition of metalloproteinase activity attenuates the loss of bone mass induced by estrogen deficiency in mice^12,13^. This evidence has suggested that suppression of *Mmp13* by estrogens may contribute to their bone protective effects. In the work presented here, we have generated mice lacking *Mmp13* in mesenchymal lineage cells to functionally interrogate the effect of the mesenchymal cell- derived MMP13 on bone and whether MMP13 produced by these cells plays a role in the loss of bone mass caused by estrogen deficiency.

## Results

### *Mmp13* expression is downregulated by ERα

**Figure 1:**
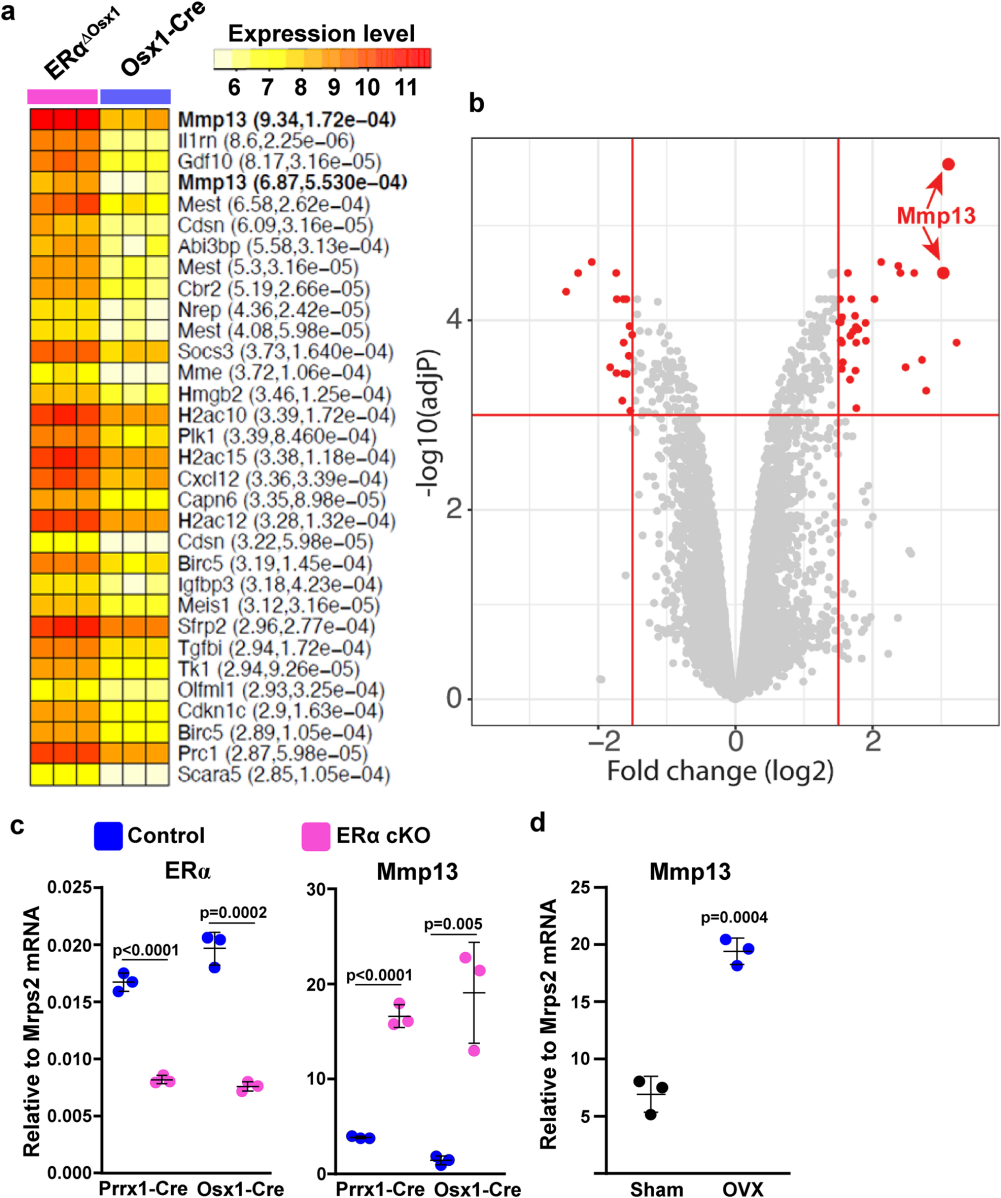
*Mmp13* is downregulated by estrogens. (**a**) Microarray analysis of GFP-sorted Osx1^+^ cells without or with ERα, derived from calvaria cells of ERα^ΔOsx1^ mice or Osx1-Cre controls. Heat map shows the normalized expression values of the top highly up-regulated genes (log2 fold change > 1.5 and adjust P values < 0.001) of individual sample. Gene names are shown on the right-side of the heatmap with fold change and adjusted p values. *Mmp13* gene, which has 2 probe sets, is in bold letters. (**b**) Volcano plots showing the profiles of differential gene expression. The gene that passed the cutoff of log2 fold change > 1.5 and adjusted P values < 0.001 are represented by red color dots. *Mmp13* gene, which has 2 probe sets is pointed to by the red arrows. (**c**) Relative mRNA levels of *ERα* and *Mmp13* in bone marrow stromal cell cultures derived from littermate control (ERα floxed) or ERα conditional KO mice (cKO) using *Prrx1-Cre;* or in calvaria cells from Osx1-Cre or ERα cKO using *Osx1-Cre*. (**d**) mRNA levels of *Mmp13* in bone marrow stromal cell cultures from ovariectomized and sham operated wild type mice (each sample is a pool of 3–4 animals from either group). Data represent mean ± S.D.; *p* values by Mann–Whitney U test.

To identify estrogen target genes that regulate osteoclastogenesis indirectly, i.e., via actions on cells of the mesenchymal lineage, we performed microarray analysis of GFP sorted Osx1 + cells without or with ERα, derived from the calvaria cells of ERα^f/f^;GFP:*Osx1-Cre* mice or GFP:*Osx1-Cre* controls. The highest up-regulated gene in ERα deleted mesenchymal/stromal cells encodes the matrix metalloproteinase 13 (*Mmp13*), as shown in the heat map (Fig. 1a) and in the volcano plot (Fig. 1b). The microarray findings from the GFP sorted ERα deleted *Osx1* + calvaria cells were confirmed by qPCR and reproduced in cultures of ERα deleted *Prrx1* + bone marrow stromal cells derived from ERα^f/f^;*Prrx1-Cre* mice (Fig. 1c). Moreover, the mRNA levels of MMP13 were fourfold higher in bone marrow stromal cell cultures from OVX C57BL/6 mice, as compared to sham controls (Fig. 1d). In line with our findings, ERα regulates the *Mmp13* promoter activity in synoviocytes^14^.

### *Mmp13* deletion in mesenchymal progenitors decrease the length of the femur and tibia

**Figure 2:**
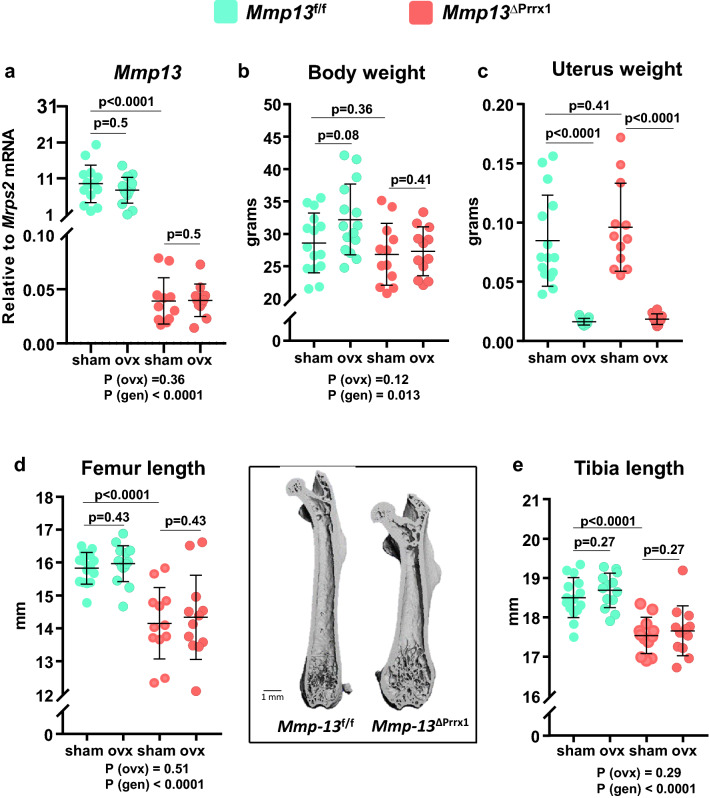
*Mmp13* deletion in mesenchymal progenitors decreases the length of both femur and tibia. Female mice with *Mmp13* deletion in *Prrx1* expressing cells (*Mmp13*^ΔPrrx1^) and control littermates (*Mmp13f.*^/f^) where either ovariectomized (OVX) or sham-operated (Sham) at 5 months of age and euthanized 8 weeks later. (**a**) *Mmp13* relative mRNA expression in femur shafts devoid of bone marrow by qRT-PCR. (**b**) Body weight and (**c**) uterine weight measured at euthanasia. (**d**) Quantification of femur length and representative micro-CT images of longitudinal femur sections of *Mmp13f.*^/f^ and *Mmp13*^ΔPrrx1^ mice and (**e**) quantification of tibia length. Data represent mean ± S.D. (n = 12–15 mice/group); *P* values by 2-way ANOVA or Kruskal–Wallis test (**c**).

To elucidate the role of *Mmp13* in bone homeostasis in vivo, we next generated mice with conditional deletion of *Mmp13* in mesenchymal progenitors expressing *Prrx1 (Mmp13*^ΔPrrx1^) and used floxed mice (*Mmp13f.*^/f^) as control. The *Prrx1-cre* transgene targets early limb bud and a subset of craniofacial mesenchymal stem cells. We did not detect a skull phenotype. All measurements were made in the femur and/or tibia. Please note that in the following description of the results, the p values from two-way ANOVA analysis are provided below each graph. The expression of the *Mmp13* mRNA in femur and tibia shafts was dramatically decreased in the *Mmp13*^ΔPrrx1^ mice (Fig. 2a), establishing the effectiveness of the deletion. Body weight and uterine weight were not affected by the *Mmp13* deletion (Fig. 2b,c). However, femur and tibia length was decreased Fig. 2d,e).

In contrast to the microarray data of Fig. 1, we did not detect a change of the mRNA levels of *Mmp13* in the OVX *Mmp13f.*^/f^ or *Mmp13*^ΔPrrx1^ mice in the osteocyte-enriched bone marrow- devoid preparations we used for this measurement (Fig. 2a). As expected, OVX increased body weight in *Mmp13f.*^/f^ mice (Fig. 2b) and decreased uterine weight in *Mmp13f.*^/f^ and *Mmp13*^ΔPrrx1^ mice (Fig. 2c). Femur and tibia length was not affected by OVX in either genotype (Fig. 2d,e).

### *Mmp13* deletion increases cortical bone and attenuates the cortical bone loss caused by OVX

**Figure 3:**
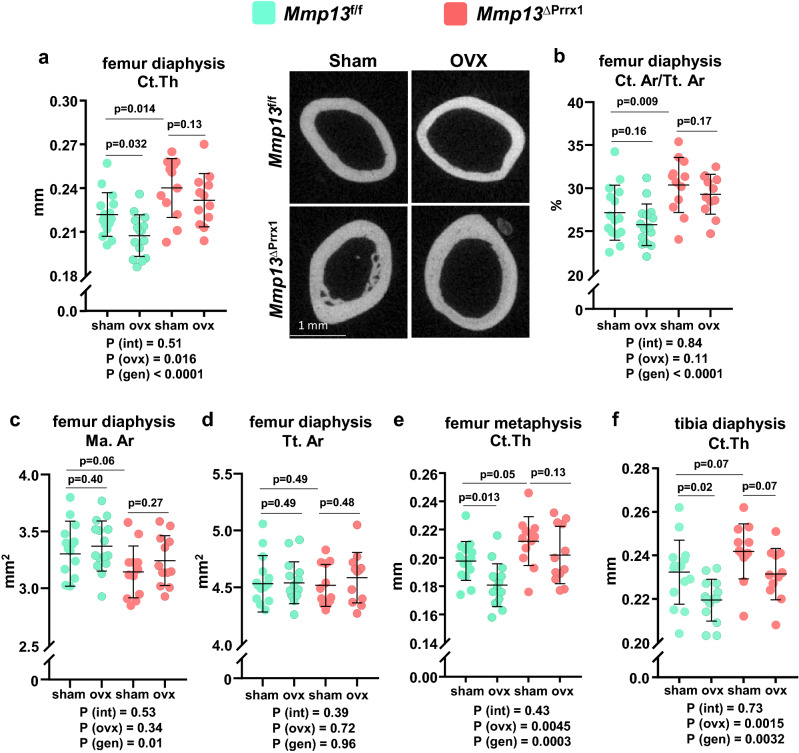
*Mmp13* deletion increases cortical bone and attenuates the cortical bone loss caused by ovariectomy. Femur and tibia cortical bone were evaluated by micro-CT. (**a**) Cortical thickness measured in diaphysis (mid-shaft) and representative micro-CT images of the same region of the femur. (**b**) Cortical area under total area, (**c**) medullary area and (**d**) total area measured in mid-shaft femur. (**e**) Cortical thickness measured at distal metaphysis of the femur. (**f**) Cortical thickness measured in the diaphysis (mid-shaft) of the tibia. Data represent mean ± S.D. (n = 12–15 mice/group); *P* values by 2-way ANOVA, interaction terms are shown below each graph.

*Mmp13* deletion in *Prrx1* cells caused greater cortical thickness and cortical area in the femoral diaphysis as indicated by pairwise comparison between sham-operated *Mmp13f.*^/f^ and sham-operated *Mmp13*^ΔPrrx1^ mice (Fig. 3a,b). This effect was due to a smaller medullary area (Fig. 3c) while total area did not change (Fig. 3d). The greater cortical thickness with *Mmp13* deletion was less marked in the distal metaphysis of the femur (Fig. 3e) and in the diaphysis of the tibia (Fig. 3f).

OVX of the *Mmp13f.*^/f^ control mice decreased cortical thickness at the femoral diaphysis and distal metaphysis as well as the tibia diaphysis (Fig. 3a,e,f). Consistent with our working hypothesis, the effects of OVX on cortical bone at the diaphysis and distal metaphysis of the femur and tibia diaphysis were attenuated in the *Mmp13*^ΔPrrx1^ mice (Fig. 3a,e,f). Together, these data suggest that *Mmp13* deletion increases cortical bone mass in femur and tibia and prevents or attenuates the loss of cortical bone caused by estrogen deficiency.

### *Mmp13* deletion increases trabecular bone but does not affect the loss of bone caused by OVX in this compartment

**Figure 4:**
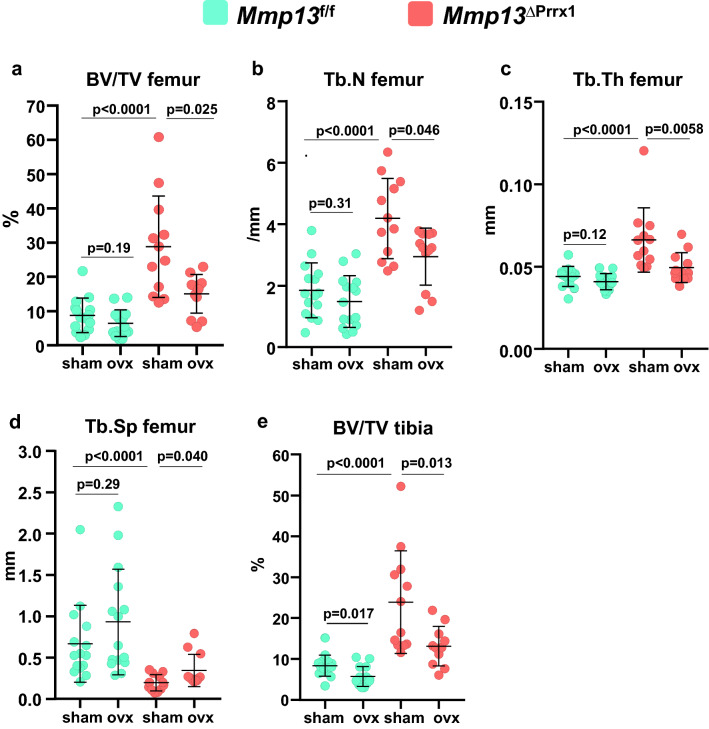
*Mmp13* deletion increases trabecular bone but does not affect the loss of bone caused by ovariectomy in this compartment. Femur and tibia trabecular bone were evaluated by micro-CT. (**a**) Trabecular bone volume and (**b**–**d**) microarchitecture at the distal metaphysis of the femur. (**e**) Trabecular bone volume at the proximal metaphysis of the tibia. Data represent mean ± S.D. (n = 12–15 mice/group); *P* values by Kruskal–Wallis tests.

Trabecular bone volume was higher in both the distal femur and the proximal tibia by approximately 3.7- and threefold respectively, in sham-operated *Mmp13*^ΔPrrx1^ mice as compared to sham-operated *Mmp13f.*^/f^ controls (Fig. 4a–e). This difference was due to higher trabecular number (Fig. 4b) and thickness (Fig. 4c); and was mirrored by lower trabecular separation (Fig. 4d). In several mice, trabecular bone extended to the midshaft (see lower left micro-CT image in Fig. 3a).

As seen before^15,16^, at 6-months of age estrogen sufficient female mice have very little trabecular bone mass remaining at the distal femur (Fig. 4a). There was no discernible effect of the OVX at this site in *Mmp13f.*^/f^ mice (Fig. 4a–d). However, we observed a loss of trabecular bone mass in both the femur and tibia of the OVX *Mmp13*^ΔPrrx1^ mice (Fig. 4a,e). Collectively, these data indicate that *Mmp13* deletion increases trabecular bone mass but does not prevent the loss of trabecular bone caused by estrogen deficiency.

### *Mmp13* deletion decreases osteoclast number in cortical bone

**Figure 5:**
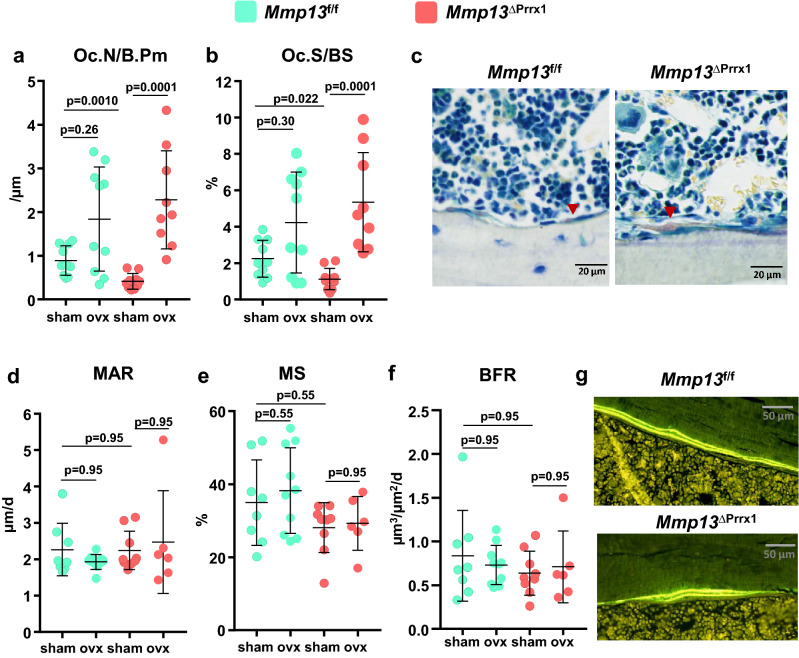
*Mmp13* deletion decreases osteoclast number at the endocortical surface of the femur. Histology and dynamic histomorphometry were evaluated at the endocortical bone surface in longitudinal undecalcified femur sections from 6-month-old female mice sham or OVX operated. (**a**) Osteoclast number per bone perimeter, (**b**) osteoclast surface per bone surface, and (**c**) representative microphotographs of osteoclasts in sections from sham animals stained with tartrate-resistant acid phosphate (*Acp5*). (**d**) Mineral apposition rate, (**e**) mineralizing surface and (**f**) bone formation rate, and (**g**) representative photomicrographs of cortical bone labeled with calcein in sections from sham animals. Data represent mean ± S.D. (n = 6–10 mice/group); *P* values by Kruskal–Wallis tests.

To elucidate the cellular mechanism by which the *Mmp13* deletion increased cortical bone volume, we performed histomorphometric analysis of the endocortical surface of the femur. *Mmp13* deletion caused an approximately 50% reduction in osteoclast number and surface (Fig. 5a–c), as indicated by pairwise comparison between sham-operated *Mmp13f.*^/f^ and sham-operated *Mmp13*^ΔPrrx1^ mice. The MMP13 deletion had no effect on mineral apposition rate (MAR), mineralized surfaces (MS) or bone formation rate (BFR) (Fig. 5d–g). These findings suggest that a decrease of osteoclast number and thereby resorption are responsible for the increase of cortical bone.

OVX of both *Mmp13f.*^/f^ and *Mmp13*^ΔPrrx1^ mice resulted in the expected increase in osteoclast number and surface (Fig. 5a,b), while MAR, MS and BFR were not affected (Fig. 5d–f). Surprisingly, in the OVX *Mmp13*^ΔPrrx1^ mice the increase in osteoclast number and surface was greater (5.5-fold) as compared to the OVX-induced increase in the *Mmp13f.*^/f^ mice (2-fold).

### *Mmp13* deletion increases whole-bone strength of the femur

**Figure 6:**
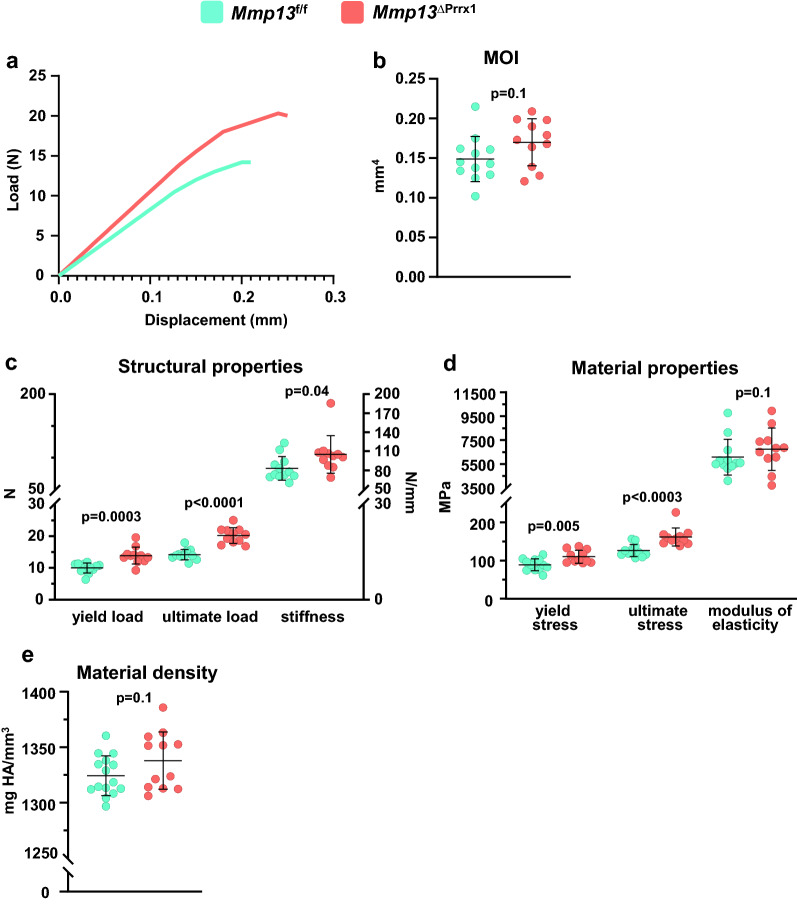
*Mmp13* deletion increase whole-bone strength in female mice. Femurs from 6-month-old *Mmp13f.*^/f^ and *Mmp13*^∆Prrx1^ female mice were tested for bone strength by 3-point bending. (**a**) Representative load *versus* displacement curve showing that femur of *Mmp13*^∆Prrx1^ mice are more resistant than control littermates. (**b**) Moment of inertia, (**c**) Structural properties, and (**d**) Material properties. (**e**) Material density by micro-CT. Data represent mean ± S.D. (n = 12–11 mice/group); *P* values by student *t* test.

It has been previously reported that *Mmp13−/−* mice have increased cortical bone fragility^17^. To examine bone strength in female *Mmp13*^ΔPrrx1^ mice we performed three-point bending of the femur (Fig. 6a). Despite the thicker cortices in female *Mmp13*^ΔPrrx1^ mice, the moment of inertia was not different from the controls (Fig. 6b). Nonetheless, the yield load, peak load, and stiffness were all higher in *Mmp13*^ΔPrrx1^ mice (Fig. 6c). With respect to the estimated material properties, female *Mmp13*^ΔPrrx1^ mice had increased yield stress and ultimate stress but the same modulus as compared to control mice (Fig. 6d). Material density determined by micro-CT was also not different (Fig. 6e). Therefore, and in contrast to a previous report, deletion of *Mmp13* led to an increase in bone structural and material properties.

### Similar to females, *Mmp13* deletion in males increases trabecular bone mass and whole-bone strength of the femur, but has no effect on cortical bone

**Figure 7:**
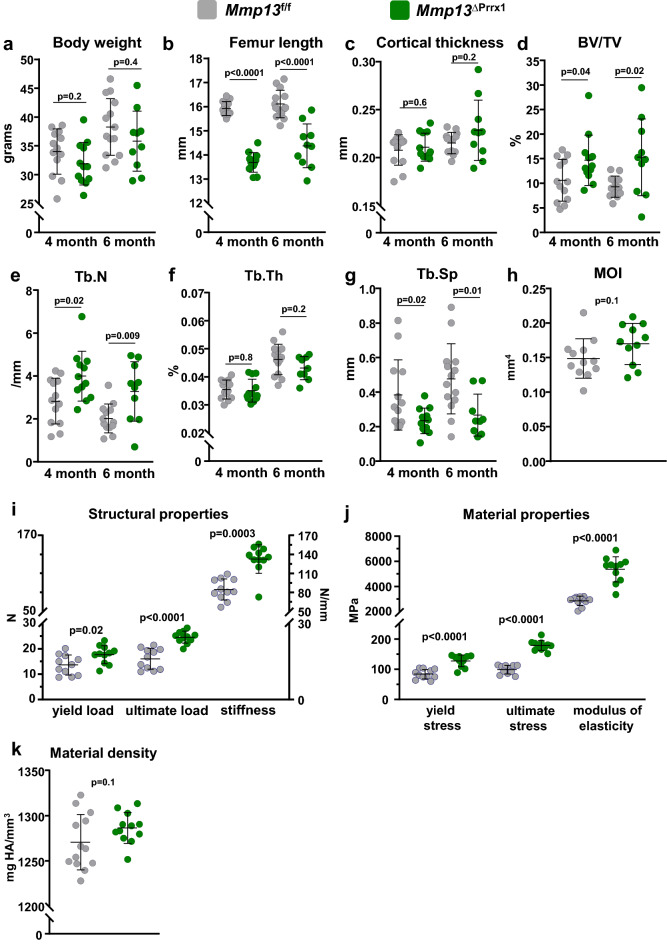
*Mmp13* deletion in males increases trabecular bone mass and whole-bone strength. Male mice with *Mmp13* deletion in Prrx1 expressing cells (*Mmp13*^ΔPrrx1^) and control littermates (*Mmp13f.*^/f^) where euthanized at 4 and 6 month of age. (**a**) Whole body weight and (**b**) femur length measured with a micrometer. (**c**) Cortical thickness at mid-shaft femur. (**d**) Trabecular bone volume and (**e**–**g**) microarchitecture at the distal metaphysis of the femur by micro-CT. (**h**–**j**) Three-point bending test in femurs from 4-month-old *Mmp13f.*^/f^ and *Mmp13*^∆Prrx1^ male mice. (**h**) Moment of inertia, (**i**) structural properties of the femurs, including yield load, ultimate load and, stiffness, (**j**) material properties of the femurs, including yield stress, ultimate stress and modulus of elasticity. (**k**) Material density by micro-CT. Data represent mean ± S.D. (n = 14–10 mice/group); *P* values by student *t* test.

The bone phenotype of sex steroid sufficient male *Mmp13*^ΔPrrx1^ and *Mmp13f.*^/f^ mice was analyzed at 4 and 6 month of age. Body weight was not affected by the *Mmp13* deletion at either age (Fig. 7a), but femur length decreased at 4 and 6 months of age (Fig. 7b), as it did in females. In difference to females, cortical thickness in male mice was not affected by the *Mmp13* deletion (Fig. 7c). Trabecular bone volume increased 1.4- and 1.6-fold at 4 and 6 months, respectively (Fig. 7d), but this increase was lower compared to the one we observed in *Mmp13*^ΔPrrx1^ female mice. The increased trabecular bone volume was associated with an increase in the number of trabeculae and a decrease in trabecular separation (Fig. 7e,g), but no change in trabecular thickness (Fig. 7f).

Finally, similar to females, three-point bending of the femur in male *Mmp13*^ΔPrrx1^ mice revealed higher bone strength including an increase in stiffness and modulus (Fig. 7h–j), with no change in material density (Fig. 7k); but unlike females the moment of inertia was decreased in males (Fig. 7h).

## Discussion

In this paper we show that the highest upregulated mRNA in mesenchymal lineage cells lacking ERα encoded MMP13; and loss of ovarian function in mice increased the expression of the mmp13 mRNA in ex vivo bone marrow stromal cell cultures. In estrogen sufficient (sham-operated) *Mmp13*^ΔPrrx1^ mice cortical thickness and trabecular bone volume in the femur and tibia were increased as compared to littermate controls, while femur and tibia length was decreased. These bone phenotypic changes were associated with a decrease in osteoclast number, but no changes in bone formation. Moreover, the loss of cortical bone caused by OVX in the femur and tibia was attenuated in the *Mmp13*^ΔPrrx1^ mice. The effect of OVX on trabecular bone, on the other hand, was not affected. These results elucidate an important role of mesenchymal cell–derived MMP13 on osteoclast number or activity, bone resorption, and bone mass. We had previously shown that the OVX-induced loss of cortical, but not trabecular bone, was attenuated in mice with conditional *Cxcl*12 deletion in *Prrx1* expressing cells. Taken together, the functional genetic evidence obtained by these two studies suggests that increased production of mesenchymal cell-derived factors, such as CXCL12 and MMP13, are important mediators of the adverse effect of estrogen deficiency on cortical, but not trabecular, bone.

The expression of MMP13 was increased in calvaria or bone marrow derived cells from ERα conditional KO mice and in ex vivo bone marrow derived stromal cell cultures from OVX mice. In contrast, loss of estrogen with OVX did not alter the expression of MMP13 in osteocyte-enriched cortical bone shafts from the femur. These findings suggest that estrogens attenuate the expression of MMP13 in stromal cells or osteoblasts, but not osteocytes. Others have shown before that the MMP13 content of osteoblastic cells in the proximal tibia increases with OVX in the rat^12^. Furthermore, in human articular chondrocytes, estradiol suppresses the expression of MMP13^18^; and an increase in MMP13 has been associated with the deleterious effects of estrogen deficiency in osteoarthritis and intravertebral disc degeneration^19^. In contrast, estrogens may promote temporomandibular joint disorders via induction of MMP-9 and MMP13 in fibrochondrocytes^20^. In synoviocytes, ERα may regulate the expression of MMP13 through the AP-1 transcriptional regulatory site^21^. However, other transcription factor binding sites such as Runx, PEA-3 and p53 in conjunction with AP-1 appear to be critical for the transcriptional regulation of *Mmp13* in other cells^22,23^, perhaps explaining the different responses of this gene to estrogens in different bone cell types.

It has been suggested before, that stimulation of collagenase activity, particularly by MMP13, acts as a coupling factor for the activation of osteoclasts^9^. However, other reports suggest that MMP13 can stimulate osteoclast activity independent of its enzymatic activity^11^. *Mmp13* null mice are resistant to the loss of bone mass caused by multiple myeloma, though the number of osteoclasts on bone was unaffected by the MMP13 deletion^24^. In co-cultures, stromal cells derived from *Mmp13* null mice increase the number of osteoclast, however these osteoclasts are smaller and resorb less bone. This evidence along with our findings that ablation of *Mmp13* in *Prrx1* expressing cells does not prevent the increase in osteoclast caused by OVX, supports the idea that in pathologic conditions MMP13 may promote the bone resorbing activity of osteoclasts.

In line with previous evidence from *Mmp13* null mice^6^, we found that *Mmp13* deletion in the *Prrx1* targeted mesenchymal progenitors decreased the length of the femur and tibia in both male and female mice. This effect most likely results from the expansion of hypertrophic cartilage in the growth plate that occurs during development and growth and it is caused by the deletion of *Mmp13* in growth plate chondrocytes^7^, which are targeted by *Prrx1*-Cre. We also confirmed herein that *Mmp13* deletion increases trabecular bone volume in the femur and tibia. This effect was seen in both sexes. However, *Mmp13*^ΔPrrx1^ females exhibited a bigger increase than males. Deletion of *Mmp13* in *Col1*-Cre or *Dmp1*-Cre targeted cells also increases trabecular bone mass, indicating that osteoblasts and/or osteocytes are the major source of MMP13 responsible for this effect^7,8^. Nonetheless, we cannot exclude the possibility that MMP13 in cells of the mesenchymal lineage other than osteoblasts and osteocytes contributes to the changes in bone mass.

In the present report we show for the first time that *Mmp13* deletion in females increases cortical thickness; and this effect may be associated with a decrease in osteoclast number and/or activity in the endocortical surface with no changes in bone formation. Interestingly, and similar to previous reports in male *Mmp13* KO mice^17^, cortical thickness was unaffected in our male *Mmp13*^ΔPrrx1^ mice. Despite the increase in trabecular bone mass, we found no changes in osteoclast number or bone formation in trabecular bone in 6-month-old females. Nonetheless, Nakatani et al.^25^ have shown that the number of osteoclasts is severely decreased in the trabecular bone of 8-day-old *Mmp13* KO mice. Thus, it is possible that osteoclast number was decreased at an earlier age in our mice. Collectively, this evidence indicates that MMP13 is a potent stimulant of bone resorption, particularly in female mice.

It has been reported previously that male mice with global *Mmp13* deletion or osteocyte-specific *Mmp13* ablation have defective osteocyte perilacunar remodeling and decreased bone toughness^17^. Albeit, the mice with the osteocyte-specific *Mmp13* ablation exhibited incongruent changes in cortical bone biomechanical properties: decreased ultimate load but increased yield load and yield stress. The *Prrx1*-Cre transgene in our *Mmp13*^ΔPrrx1^ mice has inexorably deleted *Mmp13* in osteocytes. Yet, in contrast to these previous findings, both male and female *Mmp13*^ΔPrrx1^ mice exhibited increased femoral bone strength. We have not performed an examination of the osteocyte canalicular network in our mice, but the increase in femoral strength we found in *Mmp13*^ΔPrrx1^ mice argues against a biomechanically consequential change of the lacuna-canalicular network.

Cellular senescence is a hallmark of aging^26–29^ and a state in which cells secrete an array of pro-inflammatory cytokines, chemokines and proteases, known collectively as the Senescence Associated Secretory Phenotype (SASP)^30,31^. Work by us and others has shown that osteoprogenitors and osteocytes from old mice exhibit markers of senescence and SASP, including higher levels of MMP13 and CXCL12 and that the adverse effects of aging on murine cortical bone are due, at least in part, to cellular senescence^32–34^. As shown herein and in our previously published studies with *Cxcl12*^ΔPrrx1^ mice, both MMP13 and CXCL12 contribute to the loss of cortical bone caused by estrogen deficiency. We find it intriguing that some of the same cytokines may be responsible for the increase in osteoclast number and loss of cortical bone caused by both estrogen deficiency and old age.

In conclusion, the work described herein, adds to and fully supports a long line of evidence that MMP13 plays an important role, not only in bone development, but also during bone remodeling in postnatal life. These effects are evidently mediated by changes in osteoclast number or perhaps activity during the resorption phase of remodeling. Furthermore, *Mmp13* is a target gene of ERα signaling in mesenchymal progenitors and their descendants, such as bone marrow stromal cells, osteoblast progenitors, and matrix synthesizing mature osteoblasts. Loss of the restraining effect of estrogens on MMP13 in estrogen deficient states, such as OVX in mice and menopause in women, may therefore be an important culprit of the increased resorption associated with these states. Importantly, even though mesenchymal cell–derived MMP13 influences trabecular bone mass, it plays no role in the loss of trabecular bone caused by estrogen deficiency, highlighting the striking divergence of the cellular targets and downstream mediators of the effects of ERα activation by estrogens in cortical versus trabecular bone. Whether MMP13 plays also a role in the SASP-mediated loss of cortical bone in old age needs to be functionally investigated in future studies.

## Methods

### Animal experimentation

Mice with conditional deletion of ERα using *Osx1-* and *Prrx1-Cre* and respective littermates were generated as previously described^35^. Mice with conditional deletion of *Mmp13* in the mesenchymal lineage were generated by a two-step breeding strategy. Hemizygous *Prrx1-cre* transgenic male mice (B6.Cg-Tg(*Prrx1-cre*)1Cjt/J; Jackson Laboratories, stock #5584) were crossed with female *Mmp13* floxed (^f/f^) mice (FVB.129S-*Mmp13*^*tm1Werb*^/J, Jackson Laboratories, stock # 005710) to generate mice heterozygous for the *Mmp13* floxed allele with and without the *Cre* allele. These mice were intercrossed to generate *Mmp13f.*^/f^ (used as control) and *Mmp13*^ΔPrrx1^ mice. Offspring were genotyped by PCR using the following primer sequences: TGA TGA CGT TCA AGG AAT TCA GTT T*,* wild-type, product size 572 bp, CCA CAC TGC TCG ACA TTG, mutant, product size 372 bp and GGT GGT ATG AAC AAG TTT TCT GAG C, heterozygote, product size 372 bp and 572 bp. Offspring from all genotypes were tail-clipped for DNA extraction at the time of weaning (21 days) and then group-housed with same sex littermates. Mice were maintained at a constant temperature of 23 °C, a 12-h light/dark cycle, and had access to food and water ad libitum. All mice used in these experiments were obtained from the same group of breeders in 2 consecutive breeding cycles separated by 30 days. *Mmp13f.*^/f^ and *Mmp13*^ΔPrrx1^ littermate male mice were harvested and analyzed at 16 weeks (13 *Mmp13f.*^/f^ and 12 *Mmp13*^ΔPrrx1^) and 24 weeks of age (14 *Mmp13f.*^/f^ and 10 *Mmp13*^ΔPrrx1^). Twenty-week-old female *Mmp13*^∆Prrx1^ mice and *Mmp13f.*^/f^ littermates were either OVX or sham-operated after being stratified by body weight (fifteen *Mmp13f.*^/f^ and 15 *Mmp13*^ΔPrrx1^ were sham operated; 12 *Mmp13f.*^/f^ and 12 *Mmp13*^ΔPrrx1^ were OVX). Specifically, within each genotype, mice were sorted from low to high weight values. Mice were then assigned the numbers 1 and 2, successively. All animals with the same number were assigned to the same group. Weight means and standard deviation for each group were calculated and compared by *t*-test to assure that means were similar. Surgeries were performed in the morning under sedation with 2% isoflurane, as previously described^36^. Mice were injected with calcein (Sigma-Aldrich, C0876; 35 mg/kg body weight) 7 and 3 days before euthanasia for quantification of bone formation. Animals were euthanized 8 weeks after surgery and tissues dissected for further analyses. Whole body weight was measured 2 days before surgery, before calcein injections and before euthanasia. Uterine weights were obtained to confirm depletion of sex steroids. Investigators were blinded during animal handling and endpoint measurements. No adverse events occurred during surgeries, calcein injections and harvest procedures. For bone strength measurements 13 *Mmp13f.*^/f^ and 14 *Mmp13*^ΔPrrx1^ females with 26 weeks old were euthanized and femurs were harvested. All animal experiments followed ARRIVE guidelines and approved by the animal care guidelines for the Care and Use of Institutional Animal Care and Use Committees of UAMS and the Central Arkansas Veterans Healthcare System.

### Bone imaging

Right femurs and tibias from male and female *Mmp13f.*^/f^ and *Mmp13*^ΔPrrx1^ mice were dissected, cleaned from adherent muscle and fixed in 10% Millonig’s formalin, dehydrated, and kept in 100% ethanol at 4 °C. Two female tibiae were damaged during the harvest procedure and discarded. Femur and tibia lengths were measured with a micrometer followed by micro-CT analysis using a µCT40 (Scanco Medical, Bruttisellen, Switzerland). Bones were scanned at 12 µm nominal isotropic voxel size, 500 projection (medium resolution, E = 55 kVp, I = 72 µA, 4 W, integration time 150 ms and threshold 200 mg/cm^3^), integrated into 3-D voxel images (1024 × 1024 pixel matrices for each individual planar stack) from the distal epiphysis of the femur and the proximal epiphysis of the tibia towards the mid-diaphysis to obtain a number of slices variable between 650 and 690. Cortical thickness, total and medullary area of the diaphysis were determined using 18 slices at the femur and tibia mid-shafts and cortical thickness of the distal metaphysis was determined analyzing slices 300 to 350 (counting from midshaft region). Cortical analysis was performed with a threshold of 200 mg/cm^3^. Two-dimensional evaluation of trabecular bone was performed on contours of the cross sectional acquired images excluding the primary spongiosa and cortex. Contours were drawn starting 8–10 slices away from the growth plate from the distal metaphysis towards the diaphysis of the femur to obtain 151 slices (12 µm/slice), or 8–10 slices away from the growth plate of the proximal metaphysis towards the diaphysis of the tibia to obtain 120 slices (12 µm/slice). For all trabecular bone measurements contours were drawn every 10 to 20 slices. Voxel counting was used for bone volume per tissue volume measurements and sphere filling distance transformation indices were used for trabecular microarchitecture with a threshold value of 220 mg/cm^3^, without pre-assumptions about the bone shape as a rod or plate. Micro-CT measurements were expressed in 3-D nomenclature as recommended by ASBMR standard guidelines^37^.

### Histology and histomorphometry analysis

After µCT analysis, the right femurs from female *Mmp13f.*^/f^ and *Mmp13*^ΔPrrx1^ mice were embedded undecalcified in methyl methacrylate. Calcein labels and osteoclasts were quantified on both endocortical surfaces in 5 μm thick longitudinal/sagittal sections using the OsteoMeasure Analysis System (OsteoMetrics, Inc. Atlanta, GA). Osteoclasts were stained for tartrate-resistant acid phosphatase (*Acp5*) using naphthol AS-MX and Fast Red TR salt (Sigma-Aldrich). The following primary measurements were made: bone surface (BS), single label surface (sL.S), double label surface (dL.S), inter-label thickness (Ir.L.Th), osteoclast number (N.Oc, /µm), and osteoclast surface (Oc.S, %). The following derived indices were calculated: mineralizing surface (MS, %), mineral apposition rate (MAR, µm/d), and bone formation rate (BFR, µm^3^/µm^2^/d). The referent for Oc.S, MS and BFR was BS, and for N.Oc B.Pm. During dynamic histomorphometric analysis we noticed that 5 mice were missing one of the calcein injections since no double labels were observed (2 mice in *Mmp13f.*^/f^ sham group, 1 mouse in the *Mmp13*^∆Prrx1^ sham group and 2 mice in *Mmp13*^∆Prrx1^ OVX group). These mice were excluded from the analysis. All histology measurements were made in a blinded fashion. We used the terminology recommended by the Histomorphometry Nomenclature Committee of the American Society for Bone and Mineral Research^38^.

### Bone strength

Three-point bending tests were performed at room temperature, with the posterior femoral surface lying on lower supports at a 6.6 mm span. Load was applied to the anterior femoral surface by an actuator midway between the two supports, at a constant rate of 1 mm/min to failure (ElectroForce 5500, TA Instruments, New Castle, DE). Load (N) and displacement (mm) were recorded. The moment of inertia (MOI) in the midshaft of the femur was calculated using geometry measured from µCT scans (model μCT40, Scanco Medical). Yield stress, ultimate stress, and modulus of elasticity were calculated using the mechanical testing parameters, moment of inertia, and µCT measurements.

### Cell cultures

Bone marrow stromal cells from 6-month-old mice with conditional deletion of ERα using *Prrx1-Cre* and control littermates, or C57BL/6 wild-type mice undergone sham operation or OVX were obtained by flushing the tibias and femurs. Cells from 3 mice per group were pooled and cultured in α-MEM (Sigma) supplemented with 20% fetal bovine serum (FBS) (Sigma), 1% penicillin–streptomycin-glutamine (PSG) (Sigma) and 50 μg/mL ascorbic acid (Sigma) in 10 cm culture dishes for 5–7 days. Half of the medium was replaced every 3 days. Adherent bone marrow stromal cells were then re-plated in triplicate in 12 well plates at 2 × 10^5^ cells per well with ascorbic acid and 10 mM β‐glycerophosphate (Sigma) to perform qPCR assays. Calvaria cells from 3–4 day-old *Osx1-Cre* ERα deleted mice were isolated and cultured as described previously^35^.

### RNA isolation and qPCR assay

The left femur and tibia shafts from female *Mmp13f.*^/f^ and *Mmp13*^ΔPrrx1^ mice were flushed to remove the bone marrow, cleaned from adherent tissues and frozen in liquid N_2_. Frozen shafts were pulverized with a multi-well tissue pulverizer (BioSpec Products, Inc. Bartlesville, OK, USA) and frozen in Trizol at − 80 °C. Total RNA was isolated following the Trizol reagent method (Life Technologies, 15596). RNA from cultured cells from ERα *Osx1-* and *Prrx1-Cre* deleted mice and control littermates were extracted using the same methodology. RNA was reverse-transcribed using the High-Capacity cDNA Archive Kit (Applied Biosystems, Carlsbad, CA, USA). Taqman quantitative PCR was performed to determine mRNA levels of *Mmp13 and ERa* using the Mm00439491_m1 and Mm00433148_mH primers respectively, manufactured by the TaqMan Gene Expression Assay service (Applied Biosystems). The mRNA expression levels were normalized to the house-keeping gene mitochondrial ribosomal protein S2 (*Mrsp2,* Mm00475528_m1) using the ∆Ct method^39^.

### Microarray analysis

Osx1-GFP^+^ cells were sorted in an Aria II cell sorter (BD Bioscience, San Jose, CA, USA) using the EGFP fluorochrome gate (488 nm Laser; 510/20 BP). The gating strategy used for sorting GFP positive cells was to first gate on the cell population using the FSC (forward scatter) vs SSC (side scatter) dot plot. Next, we used FSC-H (height) vs FSC-A (area) dot plot to gate out doublets. Then, we used SSC vs GFP dot plot to gate on GFP positive cells using an unstained sample as a guide to draw our positive gate. The sorted cells were harvested for RNA isolation as described above. One µg total RNA per sample was hybridized to MouseRef-8 v1 Expression beadchips (Illumina, San Diego, CA) following protocols listed on the Gene Expression and Genomics Unit of the NIA (http://www.grc.nia.nih.gov/branches/rrb/dna/index/protocols.htm). Microarray florescent signals were extracted using an Illumina BeadArray 500GX reader. The data analyses of microarray were performed in R software. The signals on each sample are preprocess and normalized using lumi package^40^. The microarray data of this study is deposited at GEO database under accession number GSE191214. Raw signal intensity files from the BeadStudio of all samples – GFP-sorted Osx1^+^ cells without or with ERα, derived from calvaria cells of ERα^ΔGFP:Osx1^ mice or GFP:Osx1-Cre controls – were processed together by lumi package^40^ under R suite software. Quantile normalization was performed to make data comparable across samples. Differential gene expression between the two groups was evaluated by moderated Student’s *t* test using limma package^41^. The statistical P values were further adjusted for multiple testing using the Benjamini–Hochberg method.

### Statistical analysis

For statistical analysis and preparation of graph plots we used GraphPad Prism 9 software, and R (v. 4.0). Data are presented as dot plots with the central line representing the mean and error bars representing standard deviation. After determining that data were normally distributed and exhibited equivalent variances among groups, mean values were compared by two-way ANOVA with Bonferroni multiple comparison test or by unpaired student t-test. When data were not normally distributed, a non-parametric method was used such as Mann–Whitney U test instead of Student’s *t* test (Fig. 1c,d). In cases where data violated ANOVA assumptions (e.g., normality) we used non-parametric Kruskal–Wallis tests and *p* values were corrected for multiple comparisons using the Benjamin-Hochberg (BH) method (Figs. 2c, 4, 5). Outliers were identified and removed by the ROUTE method with a Q = 1% or by the Grubbs test with α = 5% when data is normally distributed. Exact *p* values are shown for relevant comparisons. In line with the recommendations of the American Statistical Association, summarized by Amrhein et al.^42^, a threshold value of *p* was not used to define a statistically significant effect.

## Data Availability

The data that support the findings of this study are available from the corresponding author upon reasonable request.
